# Measures of resting-state brain network segregation and integration vary in relation to data quantity: implications for within and between subject comparisons of functional brain network organization

**DOI:** 10.1093/cercor/bhad506

**Published:** 2024-02-22

**Authors:** Liang Han, Micaela Y Chan, Phillip F Agres, Ezra Winter-Nelson, Ziwei Zhang, Gagan S Wig

**Affiliations:** Center for Vital Longevity and School of Behavioral and Brain Sciences, University of Texas at Dallas, Dallas, TX 75235, United States; Center for Vital Longevity and School of Behavioral and Brain Sciences, University of Texas at Dallas, Dallas, TX 75235, United States; Center for Vital Longevity and School of Behavioral and Brain Sciences, University of Texas at Dallas, Dallas, TX 75235, United States; Center for Vital Longevity and School of Behavioral and Brain Sciences, University of Texas at Dallas, Dallas, TX 75235, United States; Center for Vital Longevity and School of Behavioral and Brain Sciences, University of Texas at Dallas, Dallas, TX 75235, United States; Center for Vital Longevity and School of Behavioral and Brain Sciences, University of Texas at Dallas, Dallas, TX 75235, United States; Department of Psychiatry, University of Texas Southwestern Medical Center, Dallas, TX 75390, United States

**Keywords:** resting-state functional correlations, brain networks, reliability, system segregation, modularity, integration

## Abstract

Measures of functional brain network segregation and integration vary with an individual’s age, cognitive ability, and health status. Based on these relationships, these measures are frequently examined to study and quantify large-scale patterns of network organization in both basic and applied research settings. However, there is limited information on the stability and reliability of the network measures as applied to functional time-series; these measurement properties are critical to understand if the measures are to be used for individualized characterization of brain networks. We examine measurement reliability using several human datasets (Midnight Scan Club and Human Connectome Project [both Young Adult and Aging]). These datasets include participants with multiple scanning sessions, and collectively include individuals spanning a broad age range of the adult lifespan. The measurement and reliability of measures of resting-state network segregation and integration vary in relation to data quantity for a given participant’s scan session; notably, both properties asymptote when estimated using adequate amounts of clean data. We demonstrate how this source of variability can systematically bias interpretation of differences and changes in brain network organization if appropriate safeguards are not included. These observations have important implications for cross-sectional, longitudinal, and interventional comparisons of functional brain network organization.

## Introduction

Measures of network segregation are widely used to describe the topological organization of large-scale brain networks (Sporns and Betzel 2016; Wig 2017). Network segregation refers to the degree of separation between the sub-networks (also referred to as modules or communities) of a network. Network segregation can be quantified directly by calculating the relative strength of relationships within versus between sub-networks (system segregation; Chan et al. 2014) or inferred indirectly using the measure of modularity (Q) obtained via the modularity maximization algorithm (Newman 2004). In the context of brain networks, both measures are calculated from adjacency matrices created by evaluating pairwise structural or functional relationships between brain network nodes.

A large body of research has revealed that functional brain network segregation varies in relation to age, task performance, cognitive ability, and health status (for a review, see Wig 2017). In healthy individuals, segregation of resting-state functional correlation (RSFC) brain networks increases in youth and adolescence (Chen and Deem 2015; Gu et al. 2015) and declines with increasing adult age (Betzel et al. 2014; Chan et al. 2014; Geerligs et al. 2015; Han et al. 2018; Chong et al. 2019; Cassady et al. 2020; Kong et al. 2020; Madden et al. 2020; Manza et al. 2020; Stumme et al. 2020; Pedersen et al. 2021; Setton et al. 2022). Lower resting-state system segregation is associated with reduced activation selectivity across visual and frontal–parietal control areas during performance of tasks supported by these brain systems (Chan et al. 2017), worse cognitive and motor function (Chan et al. 2014; King et al. 2018; Varangis et al. 2019), and socioeconomic disadvantage (Chan et al. 2018; Chan et al. 2021). Measures of brain network segregation also have relevance toward characterizing patient populations. For example, baseline brain network modularity is predicative of rehabilitation outcomes in aphasic patients (Duncan and Small 2016), and brain network modularity changes (increases) during recovery from stroke (Siegel et al. 2018). Among Alzheimer’s disease (AD) patients, individuals diagnosed with dementia have lower modularity compared to healthy “controls” (Brier et al. 2014), increasing AD dementia severity Is associated with lower system segregation, independent of age and amyloid burden (Zhang et al. 2023), and system segregation moderates the effect of tau deposition on cognitive impairment (Ewers et al. 2021). Finally, longitudinal measurement of healthy adults indicates that declining system segregation is prognostic of dementia severity, independent of atrophy, AD genetic risk, and baseline biomarkers of AD-related brain pathology (Chan et al. 2021).

As highlighted above, the measurement of network segregation has become a popular target for researchers investigating properties of brain network organization in healthy and diseased individuals, in part because the approach offers a straightforward and objective method of effectively summarizing complex patterns of meso-scale brain network topology using a single measurement value. This value is often treated as a modifiable brain network “trait” for an individual, which allows for cross-sectional comparisons (e.g. across age, cognitive ability, or psycho-social characteristics), evaluation of longitudinal changes within individuals (e.g. development and aging), and determination of the impacts of an intervention on an individual’s brain network organization (e.g. following brain stimulation, pharmaceutics, cognitive training).

A central assumption throughout all these applications is that measures of network segregation (and other measures of network organization more generally) are a reliable index of an individual’s network topology. This requirement is in turn partly predicated on obtaining reliable RSFC relationships between distributed brain locations, which has been previously examined using specific seed-based correlation maps (Van Dijk et al. 2010; Shah et al. 2016; O'Connor et al. 2017) or specific ICA patterns (Zuo et al. 2010; Zuo and Xing 2014). These reports have demonstrated that reliability of RSFC can vary across different brain regions and sets of relationships between brain regions. In keeping with this, measures quantifying whole-brain RSFC patterns (Shehzad et al. 2009; Birn et al. 2013; Noble et al. 2017), multivariate analysis (Zuo et al. 2019; Noble et al. 2021), or summary measures of RSFC network topologies (e.g. small-worldness [Braun et al. 2012], modularity and clustering coefficient [Cao et al. 2014], and degree centrality [Chen et al. 2015]) have been shown to be generally more reliable than single pair-wise correlations or correlation maps. However, even among these studies there is a considerable range of measurement reliability, some of which relates to differences in data acquisition (e.g. eyes open vs eyes closed, awake vs. asleep), test-retest intervals, data pre-processing decisions, and topological characteristics of the examined RSFC relationships (for reviews, see Noble et al. 2019; Zuo and Xing 2014).

One source of variability in these preceding observations likely relates to the amount of data used to measure an individual’s RSFCs (Birn et al. 2013). Measures of RSFC network topography and topology can vary in relation to data quantity used to calculate the measures when resting-state data are combined across multiple sessions (Laumann et al. 2015; Gordon et al. 2017). These important observations indicate that the inter-session reliability of RSFC network measures may also vary as a consequence of data amount. Critically, the amount of data used to calculate an individual’s functional network organization can vary across studies, as well as across participants within a study, either due to variability in data acquisition or variability in the effectiveness of preprocessing methods used to minimize known sources of non-neuronal variance.

Why would variable amounts of data contribute toward obtaining valid and reliable estimates of measures of network organization for an individual? There exist multiple time-varying sources of information that contribute variance to resting-state signals. RSFC has been shown to vary over time, both when evaluating different scans collected in close proximity (Honey et al. 2009; Liu et al. 2009; Shehzad et al. 2009; Van Dijk et al. 2010), and also on much shorter timescales within a single scan (from seconds to minutes) (Chang and Glover 2010; Sakoglu et al. 2010; Kiviniemi et al. 2011; Handwerker et al. 2012; Jones et al. 2012). It has been proposed that the temporal fluctuation of resting-state signals relates to changes in ongoing cognitive processing (e.g. Esposito et al. 2006; Fransson 2006; Sun et al. 2007; Fornito et al. 2012; Gratton et al. 2018), outcomes of learning (Albert et al. 2009; Lewis et al. 2009; Stevens et al. 2010; Tambini et al. 2010; Bassett et al. 2011), and transitions between states related to sleep (Horovitz et al. 2008; Horovitz et al. 2009) or level of arousal (Tagliazucchi et al. 2013; Becker et al. 2016). However, a considerable portion of both the moment-to-moment and day-to-day RSFC variability can be attributed to data sampling error, head motion, and fluctuating drowsiness (Laumann et al. 2017). Importantly, efforts to alleviate the described sources of non-neuronal variance include truncation of time series (e.g. “frame scrubbing” [Power et al. 2020]), or inclusion of regressors that tag moments which can vary in their number across individuals (e.g. “spike regression” [Satterthwaite et al. 2013]). These processing steps are necessary for mitigation of sources of variance of non-interest (Ciric et al. 2017), but they typically result in both less data and variable amounts of “clean” data across scan sessions within and/or between participants.

Altogether then, it is clear that (i) measures of RSFC network organization can vary at shorter timescales, and (ii) that the efforts to “clean” functional data can result in a variable amount of data used to estimate measures of functional relationships across individuals, or within individuals when comparing different scan sessions. This poses independent but related challenges for using measures of RSFC network segregation as an individual’s brain network trait and for assessing the reliability of the measures. Do measures of brain network segregation vary relative to the amount of data used to derive them? If so, data cleaning techniques can introduce potential sources of variance related to data quantity. Further, it is critical to determine the extent to which within-individual variability is small enough such that the network estimates are deemed reliable across independent scans/sessions.

In the current report, we examine the extent to which measures of brain network segregation vary in relation to data quantity used to derive the adjacency matrices from a given scanning session for an individual, and whether the measures are reliable across independent scan sessions for that individual. Based on these observations, we highlight several practical issues for the analysis of cross-sectional, longitudinal, and interventional comparison, and offer means of addressing them. To achieve these aims, we use both cross-sectional and multi-session datasets comprising multiple independent cohorts of individuals that range in age from younger to older adulthood (21–100 y).

We primarily focus on measures of brain network segregation throughout this report (i.e. system segregation [Chan et al. 2014; Wig 2017] and modularity [Newman 2004; Rubinov and Sporns 2010]). However, given their close relationship to measures of network segregation, we also include related measures of brain network integration to evaluate their sensitivity to data amount (specifically, mean clustering coefficient and mean participation coefficient [Guimera et al. 2005; Watts and Strogatz 1998]). While our concentration is on patterns of resting-state correlations obtained using blood oxygen level dependent (BOLD) imaging, the conclusions are also relevant for quantifying other types of functional relationships (e.g. during task performance or passive viewing of dynamic stimuli such as movies) and other modalities of data acquisition that involve estimating time-series relationships [e.g. electroencephalography (EEG), magnetoencephalography (MEG)].

## Materials and methods

### Datasets

Three datasets were analyzed in this report: data from the Midnight Scan Club (MSC; Gordon et al. 2017), Human Connectome Project Young Adult (HCP-YA; Van Essen et al. 2013), and the Lifespan Human Connectome Project Aging (HCP-A; Bookheimer et al. 2019). The first dataset was used for the main analyses and the latter two were used for the purpose of demonstrating the implications of the findings.

#### Midnight Scan Club

The MSC dataset comprises data from 10 participants (5 females; age range: 24–34 y, *M* = 29.1 y, SD = 3.3). These data are publicly available (https://openneuro.org/datasets/ds000224/). One participant was excluded from this study due to excessive sleep and head motion (Gordon et al. 2017). The scanning protocol was approved by the Washington University Institutional Review Board and School of Medicine Human Studies Committee. All participants provided written informed consent.

#### Human Connectome Project Young Adult

The dataset includes data collected from 1,206 participants (age range: 21–35 y; HCP s1200 release dataset; Glasser et al. 2013; Marcus et al. 2013). This release has been made publicly available (http://www.humanconnectome.org). The scanning protocol was approved by the Washington University in St. Louis’s Human Research Protection Office and all participants provided written informed consent. Participants with at least 20 min of clean resting-state data per session (see RSFC Preprocessing) were included in the final sample [*n* = 730 (*n*_female_ = 410), age range: 22–35 y, *M* = 28.6 y, SD = 3.6].

#### Lifespan Human Connectome Project Aging

The dataset includes data collected from 689 participants (age range: 35–100 y; HCP-A release 1.0 dataset [https://nda.nih.gov]). The scanning protocol was approved by the Washington University in St. Louis’s Human Research Protection Office and all participants provided written informed consent. Participants with at least 20 min of clean resting-state data total (see RSFC Preprocessing) were included in the final sample [*n* = 535 (*n*_female_ = 314), age range: 36–100 y, *M* = 57.9 y, SD = 14.8].

### Imaging data acquisition

#### Midnight Scan Club

Each participant’s brain images were collected on a Siemens TRIO 3 T MRI scanner from 12 sessions on separate days.

##### Anatomical images

Across two separate days, four T1-weighted images (TR = 2,400 ms, TE = 3.74 ms, TI = 1,000 ms, flip angle = 8°, 224 slices) and four T2-weighted images (TR = 3,200 ms, TE = 479 ms, resolution = 0.8 × 0.8 × 0.8 mm^3^, 224 slices) were obtained for each participant.

##### Functional images

Resting-state functional MRI images were acquired across 10 subsequent days (one session per day). In each session, 30 min of resting state fMRI data were collected, in which participants visually fixated on a white crosshair presented against a black background. All functional imaging was performed using a gradient-echo EPI sequence (TR = 2,200 ms, TE = 27 ms, flip angle = 90°, resolution = 4 × 4 × 4 mm^3^, 36 slices).

#### Human Connectome Project

Participants were scanned on a Siemens 3 T Prisma whole-body scanner (Siemens, Erlangen, Germany) with a Siemens 32-channel head coil at one of 4 different sites (Massachusetts General Hospital, University of California-Los Angeles, University of Minnesota, and Washington University in St. Louis). Each participant completed two scanning sessions on two separate days.

##### Anatomical images

Each participant has two T1-weighted magnetization-prepared rapid acquisition gradient echo (MP-RAGE) structural scans (TR = 2,500 ms, TE = 2.1 ms, TI = 1,000 ms, resolution = 0.7 × 0.7 ×  0.7 mm^3^, flip angle = 8°) and two T2-weighted structural scans (TR = 3,200 ms, TE = 563 ms, resolution = 0.7 × 0.7 × 0.7 mm^3^).

##### Functional images

Resting-state functional MRI images were acquired while the participants fixated on a white crosshair on a black background using a gradient-echo EPI sequence (multiband factor = 8, TR = [HCP-YA: 720 ms; HCP-A: 800 ms], TE = [HCP-YA: 33.1 ms; HCP-A: 37 ms], flip angle = 52°, 104 × 90 matrix size, 72 slices, 2 mm isotropic voxels, and 1200 time points [14.4 min] per scan for HCP-YA, and 688 time points [6.5 min] per scan for HCP-A). There were two resting-state scans in each scan session, with different phase-encoding directions (HCP-YA: RL and LR; HCP-A: AP and PA) in each scan.

### Processing of anatomical MRI image and cortical surface

#### Midnight Scan Club

For each participant, their mean T1- and T2-weighted images were derived by averaging multiple structural images of the same modality, then underwent distortion correction using the mean field map (Laumann et al. 2015; Gordon et al. 2017). The following steps were taken to generate cortical surfaces: (i) generating anatomical surfaces from the participant’s average T1-weighted image in native volumetric space using FreeSurfer’s (version 5.3) default recon-all processing pipeline; (ii) registering the fsaverage-registered surfaces from FreeSurfer pipeline using deformation maps from a landmark-based registration of fsaverage surfaces to a hybrid atlas surfaces (fs_LR; Van Essen et al. 2012a) and resampling to a resolution of 163,842 vertices (164 k fs_LR) using Caret tools (Van Essen et al. 2001); and (iii) down-sampling each participant’s 164 k fs_LR surfaces to a 32,492 vertex surfaces (fs_LR 32 k). The deformations from the original surfaces to the fs_LR 32 k surfaces were composed into a single deformation map allowing for a one-step resampling.

#### Human Connectome Project

Anatomical MRI images were processed using HCP structural pipelines that consist of 3 parts (PreFreeSurfer, FreeSurfer and PostFreeSurfer). The PreFreeSurfer pipeline is used to produce an undistorted native structural volume space for each participant, align the T1- and T2-weighted images, perform a B1 (bias field) correction, and register the participant’s native structural volume space to MNI space. The FreeSurfer pipeline (HCP-YA: version 5.3; HCP-A: version 6.0) segments the volume into predefined structures, reconstructs white and pial cortical surfaces, and performs FreeSurfer’s standard folding-based surface registration to their surface atlas (fsaverage). The final structural pipeline (PostFreeSurfer) produces all of the NIFTI volume and GIFTI surface files, along with applying the surface registration to the Conte69 surface template (Van Essen et al. 2012a), down-sampling registered surfaces, creating the final brain mask, and creating myelin maps.

### Basic fMRI preprocessing

#### Midnight Scan Club

The MSC dataset was preprocessed with slice-timing correction (odd vs. even slice due to interleaved acquisition), intensity normalization to a whole brain mode value of 1,000 (Miezin et al. 2000), and realignment within-run to correct for head movement. BOLD data were transformed to Talairach atlas space by registering the mean intensity image from a single BOLD session to the atlas (Talairach and Tournoux 1988) using the average high-resolution T1- and T2-weighted images. All other BOLD sessions were linearly registered to the single atlas registered BOLD session. The atlas transformation, mean field distortion correction, and resampling to 3-mm isotropic atlas space were combined and performed via a single interpolation.

#### Human Connectome Project

Both HCP datasets employed the HCP fMRI volume pipeline to correct gradient-nonlinearity-induced distortion, realign the time series to correct for head motion, perform EPI fMRI image distortion correction due to phase encoding directions, and combine all the transforms for each registration and distortion correction steps into a single nonlinear transformation that can be applied in a single resampling step. After transforming the fMRI volumes, the intensity across runs was normalized to a whole brain mode value of 1,000 (Miezin et al. 2000).

### RSFC preprocessing

For all three datasets, additional preprocessing steps were taken to reduce spurious variance unlikely to reflect neuronal activity in RSFC data (Power et al. 2014): (i) demeaning and detrending; (ii) multiple regression of the BOLD data to remove variance related to the whole brain gray matter signal (global signal regression; defined by each participant’s own anatomy), ventricular signal, white matter signal, six detrended head realignment parameters obtained by rigid-body head motion correction, and the first-order derivative terms for all aforementioned nuisance variables. While the use of global signal regression in RSFC processing has attracted varying opinions, it is objectively effective for minimizing motion-related artifacts (Satterthwaite et al. 2013; Power et al. 2017), and the recommended method to reliably remove global cardiac- and respiration-related artifacts when direct estimates of these signals are unavailable (Power et al. 2018). Because older adults are more prone to head movement (Van Dijk et al. 2012; Savalia et al. 2017) that lead to altered RSFC profiles [Satterthwaite et al. 2013; Power et al. 2014], it is critical to minimize this source of bias that may contribute to erroneous estimation of RSFC. (iii) To reduce the effect of motion artifacts on RSFC, data were processed following a “scrubbing” procedure (Power et al. 2014). Motion-contaminated volumes were identified by frame-by-frame displacement (FD) that was calculated as the sum of absolute values of the differentials of the three translational motion parameters and three rotational motion parameters (Power et al. 2014). Volumes with excessive head motion (FD ≥ 0.2 mm for MSC) were flagged, based on previous demonstrations of the effectiveness of this FD cutoff for this dataset (Gordon et al. 2017). Recent studies have demonstrated that high-frequency respiratory artifact can confound estimates of FD, particularly for scans using multiband sequences with short TRs (e.g. Fair et al. 2020). As such, the motion parameters for HCP datasets were filtered to remove high-frequency components prior to the estimate of FD and more stringent cutoffs for FD were used for HCP-YA (0.04 mm; Gordon et al. 2017) and HCP-A datasets (0.08 mm; as has been used for analyzing datasets with wider age-range and shorter total scan duration; Gratton et al. 2020). In addition, for all datasets, data between two motion-contaminated frames that were fewer than five frames apart were also flagged. These flagged motion-contaminated frames were removed and interpolated for the subsequent processing. (iv) Band-pass filtering (0.009 < *f* < 0.08 Hz). (v) Removing the interpolated frames that were used to preserve the time series during regression and bandpass filtering.

### CIFTI generation

Connectivity Informatics Technology Initiative (CIFTI) format files were generated to integrate information from all possible brain ordinates, including the cerebral cortex, subcortical structures, and the cerebellum. To do so, the time series data of the cortical surface were derived by resampling functional volumes to 32 k mesh surface using single deformation maps derived from surface data processing and smoothed with a Gaussian kernel (MSC: 6 mm full width half-maximum [FWHM]; HCP: 2 mm FWHM). The subcortical data were smoothed in volumetric space with a Gaussian kernel (MSC: 6 mm FWHM; HCP: 2 mm FWHM). Finally, the time-series data of the cortical surface and volumetric time series of FreeSurfer-labeled subcortical structures and the cerebellum were combined to create CIFTI files (Glasser et al. 2013).

### Data sampling

**Fig. 1:**
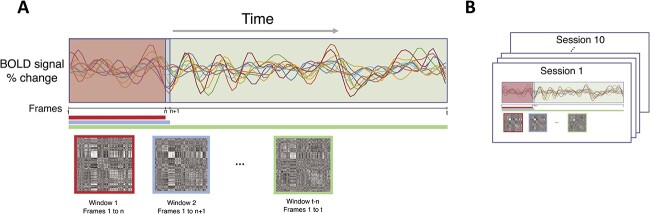
To examine impacts of data quantity, individuals’ resting-state data were sampled using expanding windows for each of their scan sessions. (A) Within a given session for a participant, we began by sampling only the 1st–*n*th frames needed to obtain 1 minute of data (window 1; e.g. for the MSC dataset this is the 1st–28th frame given a 2.2 s TR), followed by the 1st to *n* + 1 frames (window 2) and so on, successively adding a single additional frame until all frames were included. The participant’s correlation matrices and resting-state network measures were calculated separately for each window, thereby estimating the participant’s brain network organization across an increasing range of data amounts. (B) The process in (A) was repeated for each of the participant’s independent scan sessions.

To estimate the impact of including different amounts of data on estimating brain networks, expanding windows were used to sample frames from the entire functional time series in each session of a participant. Specifically, we started by sampling from the 1st frame to the *n*th frame needed to obtain 1 minute of data to form the first window (Window 1 = 28 frames of data for MSC dataset [TR = 2.2 s]), then used this data window to compute the participant's correlation matrix and corresponding network measures. This size of this data window was incremented by one and the process was repeated until all frames were sampled (i.e. 1st–*n*th frames, 1st–*n*th + 1 frames, …, 1st–*t* frames; *t* = total number of frames in each session; Fig. 1).

### Brain network construction and estimation

#### Brain network nodes

For the analysis using MSC data, individual parcellations were used to define nodes for the construction of RSFC correlation matrix (Gordon et al. 2017), given that each participant had a substantial amount of data available, making individual parcellations more reliable (Laumann et al. 2015). In the validation analyses using HCP datasets, we used a previously published atlas node set derived from an independent group of participants (Gordon et al. 2016). The reasoning for using a parcellation atlas in these cases was that the parcellation from a large pool of participants with high-quality data can capture important features of brain network organization in alternate datasets (Power et al. 2011; Yeo et al. 2011; Wig et al. 2014), which would also ensure generalization of the findings.

#### Constructing brain networks

For each data-sampling window, the functional time series were averaged across all the vertices within each node, resulting in an *n* × *t* matrix (where *n* is the number of nodes, and *t* is the total number of frames in this window). The time series of each node was correlated with the time series of every other node using Pearson’s correlations, and Fisher’s *r*-to-*z* transformed to generate a node-to-node correlation matrix (an *n* × *n* network graph matrix). Following previous reports (Chan et al. 2014; Han et al. 2018; Chan et al. 2021), negative correlations were excluded in the brain network graph matrix (negative values were set to 0). Network measures were calculated from these correlation matrices.

#### Community assignments

In the primary analysis using the MSC dataset, network communities of the node-wise graphs were identified using the Infomap algorithm (Rosvall and Bergstrom 2008). Specifically, each participant’s network graph matrix in a data-sampling window was (i) thresholded at each edge density ranging from 0.3 to 5% in steps of 0.1% (i.e. correlations above each threshold were retained, and correlations below the threshold were set to 0); (ii) filtered to exclude any correlations between nodes whose centroids were less than 30 mm apart (these edges were set to 0); and (iii) entered into the Infomap algorithm for the estimation of community assignments at each edge density. For the analysis in HCP datasets, we used a previously published atlas node set and community assignments (Gordon et al. 2016).

### Calculation of network measures

Two measures of brain network segregation were evaluated: (i) system segregation and (ii) modularity. In addition, two widely studied measures of brain network integration were also evaluated. Network integration reflects the degree to which information from distributed brain network nodes is communicated and combined (Rubinov and Sporns 2010); measures of network integration have typically relied on measurement of the (i) mean clustering coefficient (CC; reflecting the degree to which nodes in a network tend to cluster together) and (ii) participation coefficient (PC; reflecting intermodular connectivity between brain network nodes.) across nodes of the network.

To quantify the changes/differences of measures attributable to different amounts of data being used, network measures were computed from matrices defined for each data-sampling window.

System segregation summarizes values of within-system correlations in relation to between-system correlations, reflecting the degree to which the functional brain network is organized into distinct subnetworks (Chan et al. 2014; Chan et al. 2021). Following previous reports (Chan et al. 2014; Chan et al. 2021), negative correlations were excluded in the brain network graph matrix (negative values were set to 0). Without thresholding the correlation coefficients, system segregation takes the difference in mean within-system and mean between-system correlation as a proportion of mean within-system correlation, as noted in the following formula (Chan et al. 2021):


$$ System\ Segregation=\frac{\frac{\sum_w^W{Z}_w}{W}-\frac{\sum_b^B{Z}_b}{B}}{\frac{\sum_w^W{Z}_w}{W}} $$

where ${Z}_w$ represents the connectivity among nodes (Fisher z-transformed correlation coefficients) within the same system, ${Z}_b$ denotes the connectivity between nodes of one system and nodes of other systems, $W$ is the total number of within-system edges across all functional systems, and $B$ is the total number of between-system edges across all functional systems. In each data-sampling window, system segregation was computed based on the community assignments at each edge density (top 0.3–5%) and then averaged across all edge thresholds for MSC dataset. In the HCP datasets, system segregation was calculated using a previously published set of community labels (Gordon et al. 2016).

Modularity quantifies the degree to which the entire brain network could be divided into separate functional systems (Newman 2004):


$$ Q=\frac{1}{l}{\sum}_{i,j\in N}\left({a}_{ij}-\frac{k_i{k}_j}{l}\right){\delta}_{m_i,{m}_j} $$

where ${a}_{ij}$ is the connection between $i$ and $j$, $l$ is the number of connections, ${k}_i$ is the degree of node $i$, ${m}_i$ is the module containing node $i$, and ${\delta}_{m_i,{m}_j}$ = 1 if ${\delta}_{m_i}$ = ${\delta}_{m_j}$ and 0 otherwise. In each expanding window, modularity was computed using thresholded RSFC graph matrix at each edge density (top 0.3–5%) and then averaged across all edge thresholds.

CC averages the fraction of triangles around each node in the network, which reflects the prevalence of clustered connectivity around individual nodes (Watts and Strogatz 1998):


$$ C=\frac{1}{n}\sum_{i\in N}\frac{2{t}_i}{k_i\left({k}_i-1\right)} $$

where ${k}_i$ is the degree of node $i$, and ${t}_i$ is the number of triangles around node $i$. In each expanding window, CC was computed using thresholded RSFC graph matrix at each edge density (top 0.3–5%), and then averaged across all edge thresholds. For each participant, CC values were averaged across all nodes, to compute a mean CC for that participant’s brain network.

PC quantifies the diversity of intermodular interconnections of individual nodes (Guimera et al. 2005). A higher PC indicates that the node has connections with multiple communities in a network. PC is formally expressed by


$$ {y}_i=1-\sum_{m\in M}{\left(\frac{k_i(m)}{k_i}\right)}^2 $$

where $M$ is a set of nonoverlapping modules in the network, ${k}_i(m)$ is the number of links between $i$ and all nodes in module $m$, and ${k}_i$ is the degree of node $i$. In each expanding window, PC was computed based on the community assignments at each edge density (top 0.3–5%), averaged across all edge thresholds for MSC data. For HCP data, PC was calculated using a previously published set of community labels (Gordon et al. 2016). For each participant, PC values were averaged across all nodes, to compute a mean PC for that participant’s brain network.

### Evaluation of reliability

The reliability of network measures was evaluated by quantifying within- and between-session variability using the MSC data. To do so, we first calculated network measures as a function of data quantity (i.e. for each expanding window) for each session, resulting in an *s* × *w* matrix for each participant (where *s* is the number of sessions, and *w* is the number of expanding windows).

#### Within-session variability

The within-session variability of a network measure was assessed by calculating the first derivative of each session’s curve, to quantify the change of network measures in relation to the amount of data used to calculate the measure. Derivative values were averaged across all available sessions to produce a 1 × *w* vector for each participant, which summarized the within-session variability of the network measures in relation to data quantity, for that participant.

#### Between-session variability

The between-session variability of a network measure was assessed by calculating the standard deviation (SD) of the measure across all sessions, for a given participant. This calculation was repeated for varying amounts of data (i.e. each expanding window size, *w*), resulting in a 1 × *w* vector of SD values for each participant, which represents the cross-session variability of a network measure as a function of the amount of data used to calculate the measure.

#### Intra-class correlation of network measures

Existing studies have assessed the reliability of RSFC networks using the intra-class correlation (ICC; Shrout and Fleiss 1979). ICC takes both within- and between-subject variability into account. ICC quantifies the degree to which the data in their own class are similar to one another. ICC values range from 0 to 1, indicating different degrees of reliability. A high ICC value denotes that each participant’s network measure estimates at different time points are more similar to one another than the estimates in other participants. ICC is computed with the following formula (Shrout and Fleiss 1979):


$$ ICC=\frac{MS_b-{MS}_w}{MS_b+\left(d-1\right){MS}_w} $$

where ${MS}_b$ is the between-subject mean squared error, ${MS}_w$ is the within-subject mean squared error, and $d$ is number of time points. In this report, we computed one ICC value in each expanding window for a network measure, resulting in a row vector of ICC values quantifying the changes of ICC as a function of amount of data being used.

### Application to within and between subject comparisons

#### Within-participant comparisons

We evaluated how including unequal amounts of data from scanning sessions collected close in time can impact the estimate of within-participant “changes” in brain system segregation. This analysis utilized the HCP-YA dataset, which consists of two independent scanning sessions per individual. The 730 HCP-YA participants were first randomly assigned into 6 non-overlapping groups of 120 participants. For every individual within a given group, brain system segregation was calculated for one scanning session using 5 min of data and compared to brain system segregation calculated from that individual’s alternate scanning session using either 5, 6, 7.5, 10, 15, or 20 min of data. The order of scanning sessions (i.e. which scan session was treated as the alternate scanning session) was randomized for each individual and each comparison. The random sampling of participants into groups was repeated 10,000 times. Comparisons of system segregation across scan sessions were evaluated using paired-samples *t*-tests.

#### Between-participant comparisons

We evaluated how including unequal amounts of data across participants will impact between-participant system segregation comparisons. Using data from the HCP-A dataset, two sets of analyses were conducted to evaluate the correlation of age and brain system segregation (12 subjects were excluded from this analysis due to their outlier system segregation [< 3 SD from the mean] values calculated using 20 min of data, resulting in a final sample of 523 subjects with an age range of 36–100 y). In the first analysis, the amount of data included in the brain system segregation calculation was equivalent across participants (range = 2.4 s–20 min). This process was repeated 10,000 times to build a distribution of correlation values. In the second analysis, the amount of data per participant was set to randomly vary across participants (range = 2.4 s–20 min), before calculating the relationship between age and brain system segregation. Given that older adults exhibit more data loss due to greater head motion (Savalia et al. 2017), this analysis was conducted such that frames were removed in a pseudo-random manner that was based on participant’s age. To do so, we first calculated distributions of number of movement-censored frames defined by the participant’s age. Using the HCP-A dataset, three age cohorts were created (earlier middle-age: 35–49 y, older middle-age: 50–64 y, older-age: 65–100 y). For each age cohort, the mean and SD of available frames after motion processing were estimated across participants. As expected, frame loss was significantly correlated with age (*r* = 0.195, *P* < 0.001), indicating older adults had a greater degree of frame loss. These parameters (mean and SD) were used to construct three age-cohort specific normal distributions of frames, which were subsequently used to sample data amounts for each participant. Specifically, data were randomly sampled for each participant but sampling was constrained by the cohort-specific distribution of frames available based on their age. Using the sampled data, segregation was computed for each participant and correlated with age across all the participants. The sampling process was repeated 10,000 times, resulting in 10,000 correlation coefficients between age and system segregation.

## Results

### Measures of brain network segregation and integration vary in relation to the amount of data used to calculate brain network edges

The primary analysis of the current report was performed using MSC dataset that comprised data from nine participants (age range, 24–34 y). Each participant underwent 10 sessions of resting-state functional MRI images acquisitions, with each session occurring on a separate day (all sessions were within 7 weeks), enabling examination of cross-session (day) reliability of brain network measures for each individual.

**Fig. 2:**
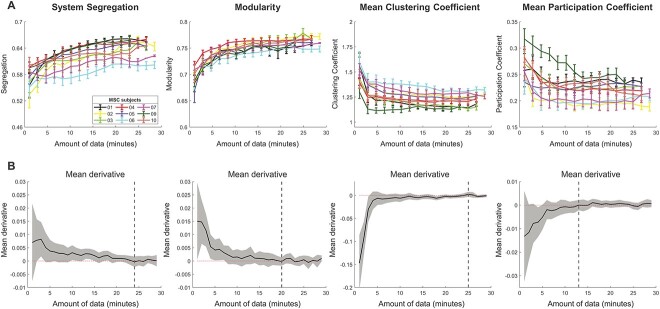
Participants’ functional network measures vary in relation to the amount of data used to construct their brain networks. Network measures were computed by sampling an increasing amount of data from a single session. (A) The resultant curves depict differences in network measure estimates as a function of the amount of data used to calculate the measure, from a given session. Curves from all sessions for a given participant were averaged to generate the curves depicted in the figure; error bars reflect within participant standard deviation across sessions for a given amount of data. Within a session, segregation and modularity increase with increasing amounts of data, whereas clustering coefficient and participation coefficient decrease. In all cases the values appear to plateau (asymptote) with larger quantities of data, and the patterns are consistent across participants. Data points are displayed in 50-frame increments (i.e. ~ 1.8 min) to facilitate visualization. (B) Participant’s functional network measures asymptote when a greater quantity of data is used to construct their brain network. The derivative of network measure curves in each session (upper panel) was calculated to quantify the change of network measures in relation to the amount of data used. Derivative values were averaged across sessions and participants. Each panel depicts the mean derivative of a network measure as a function of the amount of data used in its calculation, and the shaded area depicts the standard deviation of derivatives across all sessions of all participants. Vertical lines mark the positions at which derivative curves first reach zero and stabilize thereafter, indicating the network measures eventually asymptote with enough data. Most network measures need large amounts of data to become asymptotic (system segregation: 24 min, modularity: 20 min, mean clustering coefficient: 25 min, mean participation coefficient: 13 min).

Each of the brain network measures were computed on an adjacency matrix that was constructed with varying amount of data (in frames) used to calculate the edges of the matrix. Across data amount comparisons, frames were always sampled from the same scan session of a participant to avoid mixing within- and between-session variance (see Methods for details). We first investigated how estimates of an individual participant’s network measures varied as a function of the amount of resting-state data included. In Fig. 2(A), it is evident that both brain system segregation and modularity increase with the inclusion of more data, whereas mean CC and mean PC decrease with increasing data. The range of values can vary substantially in relation to the amount of data used to calculate the measures for a given participant. For example, system segregation values can vary up to 21.4% when comparing the measure calculated from 2.5 min of clean data to 25 min of clean data (mean difference when comparing values calculated from 2.5 min vs. 25 min, across participants and sessions: 9.12%). This observation has particularly important implications for comparisons of a participant’s brain network organization across sessions (e.g. during an interventional study examining pre- vs. post-intervention changes, or in longitudinal studies of development/aging that examine changes in brain network organization over extended durations), a point we will expand on further below.

### A participant’s network measures stabilize within a session, with sufficient amounts of data

In Fig. 2(A), it appears that each of the measures may plateau (asymptote) with larger quantities of data. Asymptotes were confirmed empirically by determining whether there exists a point at which the first derivative of the data curves reaches zero and remains stable thereafter. Figure 2(B) demonstrates that all examined network measures asymptote with sufficient amounts of data, although the asymptote necessitates a considerable amount of data and varies across measures (system segregation: 24 min, modularity: 20 min, mean CC: 25 min, mean PC: 13 min).

**Fig. 3:**
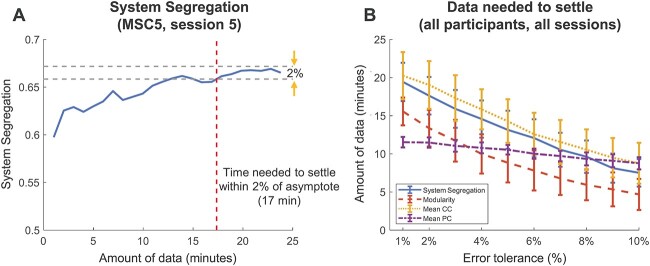
Network measures stabilize within each session for a given participant with sufficient data. (A) In an example session of an MSC participant, the settling time for system segregation is 18 min, from which the values stay steadily within 2% (error tolerance) of the final value using maximum data points. (B) The mean settling time of each network measure across all sessions and all participants using different thresholds of error tolerance (1–10%). The settling time is generally shorter if a more relaxed error tolerance is used.

A perfect asymptote may be too conservative to serve as a general criterion, and it is possible that the measures get “close enough” to their stable values with considerably less data (e.g. within 2% of their observed asymptote; Fig. 3A). Most network measures still require a considerable quantity of data to settle toward their identified asymptotic values (*M*_segregation_ = 18 ± 5 min, *M*_modularity_ = 13 ± 4 min, *M*_CC_ = 19 ± 5 min, *M*_PC_ = 12 ± 2 min; Fig. 3B). Naturally, relaxing the error tolerance relative to the stable values results in less data required for the network measures to settle.

### Stable estimates of network measures are reliable across sessions collected close in time

We have established that measures of functional brain network organization vary in relation to the amount of data used to calculate the edges of the matrices, within a scan session. These observations parallel the conclusions made in a number of previous reports (Laumann et al. 2015; Gordon et al. 2017), although, in those cases, frames were sampled randomly across multiple sessions that were aggregated across days, limiting inferences regarding within-session bias and variability.

**Fig. 4:**
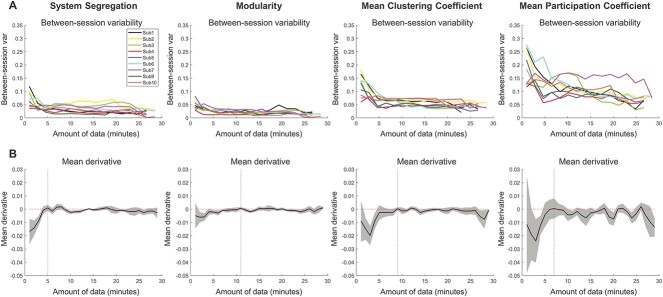
Network measures are reliable between sessions, especially when larger quantities of clean data are used in their calculation. (A) Between-session reliability was quantified by assessing the variability of measures across scans, within participants. This was calculated as the ratio of the standard deviation (SD) across sessions relative to the mean measured value. For each participant and brain network measure, the between-session variability decreases with increasing amounts of data used to calculate the brain network measure. (B) For each brain network measure, the between-session variability asymptotes with increasing amounts of data. The derivatives were calculated for each between-session variability curve in panel (A), and then averaged (black line in each panel), with the shaded area representing the standard deviation of derivatives across all sessions of all participants. Vertical lines mark the positions at which derivative curves reach zero, indicating that the between-session reliability of network measures asymptotes relatively quickly, and is high even with lesser data quantities.

Given that the value of a brain network measure can differ based on how much data is used to calculate it, it is also important to determine whether the between-session reliability of brain network measures vary in relation to data quantity. The MSC data allow for effective evaluation of measurement reliability, whereby we can test how reliable the brain network measures are across scanning sessions which have been collected close in time (within days to weeks). The between-session reliability of each network measures was quantified by assessing the variability of measures across scans, within participants. Variability was calculated as the ratio of the standard deviation (SD) across sessions relative to the mean measured value. Figure 4 demonstrates that between-session variability quickly decreases with increasing amounts of data for all network measures (critically, equivalent amounts of data were used for each session). Brain system segregation and modularity have lower between-session variability in general, relative to mean CC and mean PC.

It is evident that while the value of network measures takes a greater amount of data (time) to reach an asymptotic value (Fig. 2), considerably less data are necessary to observe high between-session reliability (Fig. 4A). This was formally evaluated by identifying the asymptotic point of the curves in Fig. 4(A). As is evident in Fig. 4(B), the between-session variability of all examined network measures asymptotes relatively quickly, although the asymptote point varies across measures (system segregation: 5 min, modularity: 11 min, clustering coefficient: 9 min, participation coefficient: 7 min).

**Fig. 5:**
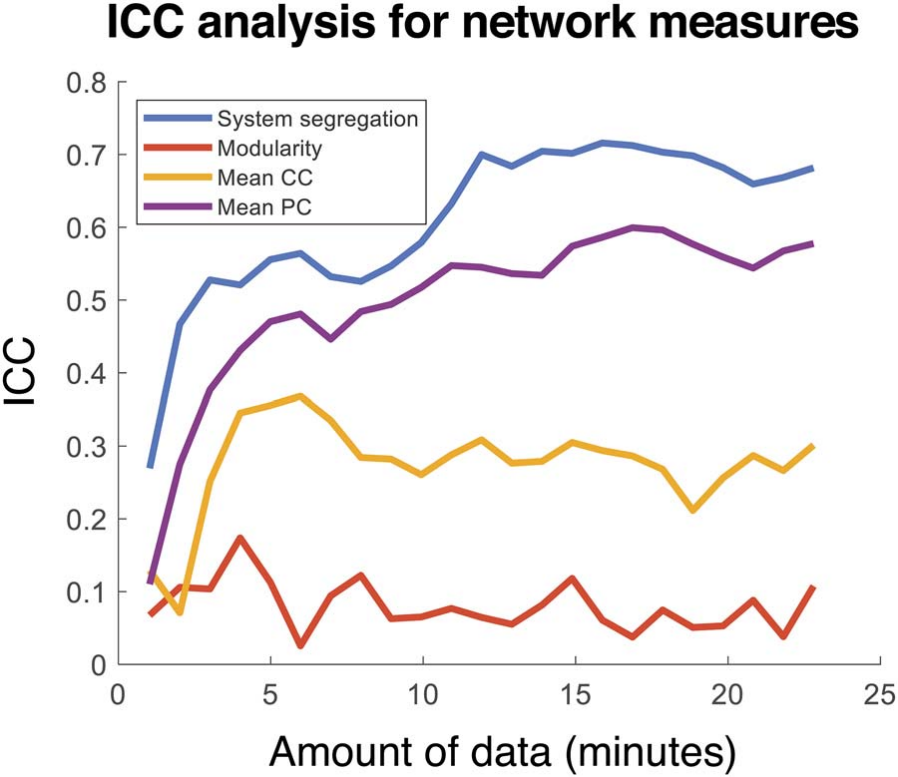
ICC of network measures varies with increasing amount of data. ICC compares between-subject variability relative to within-subject variability of each network measure. Higher ICC values indicate greater differences between participants and lesser differences within each participant across sessions. Network measures and ICC values were computed using increasing amounts of data in each session. The ICC values of system segregation and mean PC are relatively higher and continue to increase with the inclusion of greater amounts of data. ICC values of modularity and mean CC are relatively lower, and do not substantially change with greater amounts of data used to calculate the measures.

Another common way of assessing reliability of a given measure is to examine the ICC (e.g. Birn et al. 2013; Braun et al. 2012; Choe et al. 2017; Noble et al. 2017; Xu et al. 2016; Zuo and Xing 2014). ICC is a descriptive measure that takes both within- and between- subject variability into account. ICC was calculated as a function of the amount of data used for each session. The ICC values of most network measures (except modularity) increase with increasing amounts of data included per session (see Fig. 5 and Supplemental Text S1 and Fig. S1 for related observations). These values appear to plateau with about 12–15 min of data (see System Segregation and PC). ICC values for system segregation are consistently the highest, while ICC values for modularity are lowest, irrespective of the amount of data used.

Collectively, these findings reveal that measures of functional brain network segregation and integration vary with increasing amounts of data. However, both within- and between-session variability of network measures stabilize with sufficient data quantities, suggesting that reliable estimates of network segregation and integration are obtainable when enough clean data is included per participant. These results lead to important applications toward understanding the impact of data quantity on estimating network measures in cross-sectional, longitudinal, and interventional comparisons.

### Using unequal quantities of data across sessions results in artifactual estimation of brain network changes within a participant and distorts between-participant comparisons

#### Artifactual estimation of brain network changes within participants

The preceding results have demonstrated that measures of large-scale functional brain network organization vary as a function of the amount of time-series data being used in each session. This has important implications both for (i) studies that aim to compare how an individual’s network measure changes over multiple sessions (e.g. longitudinal studies of development and aging, pre/post interventional studies) and (ii) studies that aim to compare network measures across participants (e.g. in relation to age, cognitive ability, individual phenotypes, disease status, etc.). Here we demonstrate how comparing sessions with unequal amounts of data can result in misestimation of changes or differences, within or across participants, respectively.

**Fig. 6:**
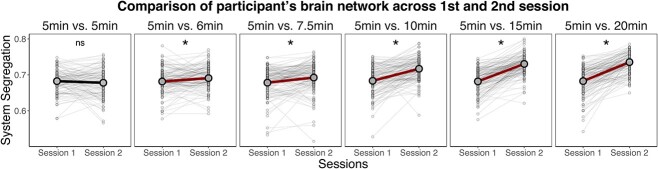
Using unequal amounts of data across scanning sessions results in systematic distortion of a participant’s change in brain network organization. HCP-YA data were collected on two consecutive days without experimental interventions, which makes this dataset a suitable candidate to examine the impact of including different amounts of data for estimating between-session differences within a participant. When the amount of data is equated across the two scan sessions, system segregation in session 1 does not differ from system segregation in session 2 (paired-samples *t*-test: *t*(1458) = −1.25, *P* = 0.212; far left panel). However, slightly varying participant’s data quantity in session 2 (6 min) leads to significantly higher system segregation values when compared to participant’s system segregation from session 1 (5 min) (*t*(1458) = 6.64, *P* < 0.001; second panel). As more data are used in session 2 relative to session 1, this between-session “change” of system segregation becomes greater (*t*s > 12.48, *P*s < 0.001). These results demonstrate that using unequal amounts of data leads to systematically biased estimates of between-session “change” of network measures within a participant. Each dot in the figure represents system segregation value of a participant; the line between each two dots shows between-session “change” within the same participant. In each plot the thick line represents the mean group change, “ns” = non-significant, “*” = *P* < 0.05.

To demonstrate the first of these problems more explicitly, we first examined a scenario where changes in an individual’s brain network organization are not expected. Specifically, we examined the HCP-YA dataset (*n* = 730, age range: 22–35 y), comparing participants’ brain system segregation across the two scanning sessions. Given that the two scanning sessions were collected in close proximity (for most participants, on two consecutive days, but at maximum a week apart, as per descriptions of the HCP-YA protocol; Van Essen et al. 2012b), and no systematic interventions were administered to participants in between the scan sessions, we expected that their brain system segregation values should remain relatively consistent across sessions. The absence of change is confirmed when the amount of data is equated across each of the participant’s scanning sessions (Fig. 6). However, Fig. 6 additionally illustrates that comparing two sessions with unequal amounts of data results in systematic inflation of the magnitude of calculated “change.” The observed “change” in brain system segregation is an artifact of failure to equate the amount of data across scan sessions and the degree of this artifact varies in proportion to the magnitude of data quantity differences across sessions. This artifact is evident even when the differences in amount of data used to calculate the brain network measure is not that large (i.e. 5 min vs. 6 min).

#### Misestimation of brain network differences across participants

The sensitivity of brain network measures to data quantity also indicates that between participant comparisons can be misestimated if appropriate steps are not taken to minimize this source of bias.

**Fig. 7:**
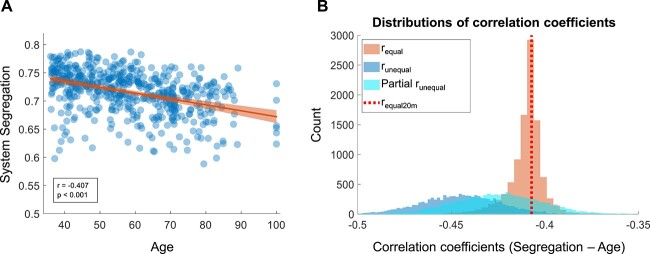
Cross-sectional relationships between system segregation and a variable of interest (in this case, age) can be misestimated using unequal quantities of data across participants. (A) System segregation computed using the maximally available amounts of equated data (20 min) across all HCP-A participants. System segregation decreases with age (*r* = −0.407, *P* < 0.001), indicating less segregated functional systems in older adults. (B) This correlation coefficient observed in (A) serves as a “standard” reference (vertical dotted line in B) for comparisons of correlation distributions. The rightmost distribution (*r*_equal_) depicts correlation coefficients between age and system segregation calculated using equal amounts of data across participants (ranging from 3 to 1500 frames). In general, the distribution is tightly distributed around the 20-min reference line. However, using unequal amounts of data tends to misestimate the relationship between segregation and age. The leftmost distribution (*r*_unequal_) depicts correlation coefficients between age and segregation calculated using unequal amounts of data across participants, whereby frame sampling was constrained in a manner that mimics the relationship between age and frame loss (see the text for details). The range of calculated correlation coefficients between age and segregation is wider, and the calculated relationships are systematically overestimated (stronger negative correlation values) than both the maximum data reference and the distribution of correlations observed when data amounts are equated across participants. The bias is still evident even when the number of frames is included as a covariate in the statistical model (the wide distribution in the middle, labeled Partial *r*_unequal_). The correlation distributions are systematically left-shifted relative to the 20-min reference line and the distribution of *r*-values when data are equated, demonstrating that this statistical maneuver does not correct misestimation of relationships between age and system segregation.

To illustrate this impact directly, we focus on the well-established relationship between increasing adult age and decreasing brain system segregation (e.g. Chan et al. 2014; for a review, see Wig 2017). To further highlight the robustness of this result, we analyzed the HCP-A dataset. Based on the preceding observations demonstrating that the measure of brain system segregation varies as a function of data quantity within participants, the number of clean frames was first equated across participants within the dataset (included HCP-A participants had 20 min of data). As expected, increasing adult age is associated with decreasing brain system segregation (*r* = −0.407, *P* < 0.001; Fig. 7A).

In practice however, data-cleaning strategies (e.g. scrubbing, frame regression) can result in an unequal number of “clean” frames that are available across participants (e.g. individuals will vary in the extent to which they move, which would result in different amounts of data remaining following scrubbing). This effect is more prominent in older adults with greater data loss due to excessive head motion (Savalia et al. 2017). This aspect of data acquisition and processing often leads researchers to include varying amounts of clean data across participants, based on the assumption that more data are better. However, as we have seen in earlier sections, data quantity can influence the estimates of network measures, so differences in data quantity across participants is potentially problematic.

To illustrate the impact of relaxing the requirement of equating frames across participants on cross-sectional comparisons, we computed correlation coefficients between age and system segregation estimated by (i) sampling a random number of frames for each participant in the HCP-A dataset (ranging from three frames [2.4 s] to 1,500 frames [20 min]), (ii) calculating system segregation for that participant, and (iii) calculating the correlation between age and system segregation across participants. Random frame sampling was constrained to mimic the relationship between age and data loss (see methods for details). This process was repeated 10,000 times, resulting in 10,000 correlation coefficients. This distribution of correlation coefficients was compared to the correlation coefficients using an equated number of frames across all participants (again ranging from 3 to 1,500 frames).

Using equal amounts of data produces greater correlation coefficients that are relatively tightly distributed (Mean = −0.409, Median = −0.348, SD = 0.014; red distribution [i.e. the rightmost distribution] in Fig. 7B) centered closely around the correlation observed when maximum data are included (red dotted line in Fig. 7B, corresponding to 20 min of data per participant as observed in Fig. 7A).

In contrast, using unequal amounts of data across participants (randomly sampled), there exists a broad spread of the correlation values (darker blue distribution [i.e. the leftmost distribution] in Fig. 7B), which are also systematically stronger than the correlation values obtained when data amounts are equated and when maximum data are used, resulting in inflated effect sizes (96.8% of the correlations are stronger than the correlation using the maximum amount of data). This observation underscores how using unequal amounts of data could significantly misestimate the relationship between segregation and age.

In some instances, investigators have opted to include number of frames as a covariate when estimating relationships between variables of interest, in order to control for potential confounds resulting from inclusion of unequal amounts of data across participants. However, Fig. 7(B) also demonstrates the inadequacy with this approach. For unequal sampling, the majority (77.8%) of partial correlations between age and segregation (in lighter blue [i.e. distribution in the middle]) are still stronger than the correlation using the maximum number of equated frames. These results demonstrate that using unequal amounts of data across participants often significantly misestimates the relationship between variables of interest in cross-sectional comparisons, even after statically controlling for the number of frames across participants.

Finally, it is important to point out that the above scenarios related to within and between participant comparisons are not rare. Unequal data comparisons for a participant and group of participants can easily be introduced from changes in imaging protocols (e.g. increasing or decreasing the number of frames or scans of resting-state data on a follow-up session), or as a consequence of data cleaning techniques that result in different amounts of clean data being used across imaging sessions. Critically, these biases are not restricted to frame scrubbing but are also evident following alternate data processing and cleaning techniques which retain all frames (see Supplemental Text S2 and Figs. S2–S5). The impacts of this source of variance are important to consider, as a systematic relationship between a given variable of interest (e.g. age of participants, patients vs. controls, time) and data quantity/quality (e.g. due to head movement changes/differences) can result in biased estimation of change that gets mis-attributed to the variable of interest.

## Discussion

The described findings demonstrate that: (i) measures of functional brain network segregation and integration are sensitive to the amount of RSFC data used to calculate each measure, although the measures asymptote with greater data quantities, (ii) obtaining reliable estimates of RSFC brain network measures within a scanning session is achievable, and (iii) the comparison of network measures within and between participants can be biased when the amount of data is not equated across scanning sessions.

### Reliable network measures are necessary for both basic science and clinical applications

Several previous studies have evaluated the reliability of RSFC maps (Van Dijk et al. 2010; Birn et al. 2013; Noble et al. 2017) and measures of RSFC networks (Cao et al. 2014; Chen et al. 2015). Reliability has been assessed with respect to various factors, including the impact of eyes open vs. eyes closed and sleep vs. awake scanning (Patriat et al. 2013), spatial differences (Van Dijk et al. 2010; Zuo and Xing 2014; Shah et al. 2016), and direct comparisons between network measures (Braun et al. 2012; Cao et al. 2014; Chen et al. 2015). While the bulk of this work has focused on data collection efforts that were more limited in terms of absolute scan length (e.g. ≤ 5 min) and number of sessions across time, they revealed weaker to modest estimates of reliability. More recent studies examining RSFC reliability have included data with greater scan lengths and sessions (e.g. O'Connor et al. 2017; Noble et al. 2019). These latter studies align with the observations presented here, demonstrating that more reliable measures of RSFC patterns are achievable with sufficient data quantity. The availability of data collection efforts that include longer lengths of resting-state data acquisition and multiple scan sessions offers the opportunity to evaluate whether, and how, measures of functional network organization vary as a function of data quantity and what the resultant reliability of these measures is. In keeping with this, we demonstrated that the reliability of network measures varies in relation to within-session data quantity, but between-session variability quickly decreases especially when data amounts are equated across sessions. This latter point underscores that in addition to considering the amount of data necessary to achieve a low degree of variability, the comparison of measures of brain network segregation and integration estimated with unequal amounts of data across sessions could bias cross-sectional, longitudinal, and interventional comparisons in relation to variables of interest. This influence of varying data amounts persists even after controlling for amount of data in the models using participant-level covariates.

Although we have focused our efforts on network measures that are defined by graph analysis, the same impacts of data equating likely apply to the estimations of node-to-node correlation values and correlation maps, given that they are used as inputs to the network measures. Likewise, the implications extend to alternate analytic approaches that examine and compare functional correlation patterns across individuals and groups of individuals (e.g. finger-printing [Finn et al. 2015; Gratton et al. 2018; Mars et al. 2018], machine-learning based prediction [Dosenbach et al. 2010; Khosla et al. 2019], gradients of connectivity patterns [Margulies et al. 2016; Huntenburg et al. 2018], and parcellation of cerebral cortex [Wig et al. 2014; Eickhoff et al. 2018]). It is likely that equating frames of data during the formation of the network matrix within and across participants is important for accurately estimating and evaluating brain network organization in general.

RSFC system segregation varies in relation to age and cognitive performance, but has also been used to differentiate unhealthy populations from control participants, which has seeded the idea of incorporating this measure in clinical applications (for a review, see Wig 2017). For example, differences in system segregation (and other related measures) are evident in adult individuals with AD (Ewers et al. 2021; Zhang et al. 2023), epilepsy (Pedersen et al. 2015), schizophrenia (Yang et al. 2016), and younger individuals with ADHD (Mills et al. 2018), suggesting that these disorders may be associated with altered “meso-scale” organization of large-scale resting-state brain networks. Among older individuals classified as “healthier,” declining system segregation has been shown to be prognostic of dementia up to 10 y prior to cognitive impairment, and independent of AD-related genetic risk, the presence of baseline AD-associated pathology and longitudinal structural changes (Chan et al. 2021). Altogether, these observations highlight the potential of applying system segregation as a biomarker of disease diagnosis and/or incipient cognitive decline. At a basic level, this application requires that the measure is a reliable descriptor of an individual’s brain network organization. Our report takes steps toward establishing this criterion by evaluating both the measurement values and the between session measurement reliability as a function of data amount. First, with sufficient data quantity in a scanning session, the system segregation values asymptote. This observation is in line with previous reports that have examined several related network measures (although not specifically system segregation; Laumann et al. 2015; Gordon et al. 2017). In addition, in the present report, system segregation was evaluated within individual scan sessions, which allowed interpretation of the impact of data quantity on measurement values as it relates to within-session estimates, an approach which is pragmatically more likely and feasible for application purposes. Second, despite the influence of data amount on measurement values, system segregation was found to be reliable across measurements obtained on multiple days as long as data amounts were equated across sessions, suggesting that the measure can be used to reliably describe the organization of large-scale functional brain networks within individuals. One notable feature was that system segregation was found to exhibit greater participant discernability than modularity, with the latter achieving notably lower ICC values irrespective of data amount (Fig. 5), indicating that system segregation may be a better individual indicator of the community-level organization of RSFC networks.

### Exploring the sources of diminished reliability and methods to alleviate them

The current study indicates that RSFC network measures vary with varying amounts of data, which may be explained from the perspective of sampling error. Using insufficient data could lead to greater sampling error that misestimates functional networks. Conversely, when more clean data are used, the degree of sampling error is minimized, which results in better estimation of the network measures. To test this hypothesis, we conducted an additional analysis where noise was directly added to the resting-state data to simulate the scenario of varying sampling error on functional network estimation; the result demonstrates that system segregation decreases with increasing noise (greater sampling error) in a manner that parallels the differences in data quantity observed throughout this report (Supplemental Text S3 and Figs. S6–S7).

Toward the previous point, in order to improve accurate estimation of resting-state functional relationships, it is critical to include data cleaning techniques that remove variance related to head motion, cardiac-signals, breathing, and other non-neuronal signals (Power et al. 2020). There exist multiple approaches that are reasonably effective toward achieving this aim. Data censoring (“scrubbing”) accompanied with global signal regression, has been argued to be an important step toward collectively minimizing distance-dependent covariance that are caused by multiple sources of noise that cannot be removed by other data cleaning techniques (Ciric et al. 2017; Power et al. 2020). The present results may lead one to conclude that data censoring is less appealing due to the introduction of variable data quantity across participants/scans. However, additional supplementary analyses indicate that other data processing techniques, including those relying on frame regression (“spike regression”) (Satterthwaite et al. 2013; Yan et al. 2013) and ICA-based regression (Beckmann et al. 2009; Nickerson et al. 2017) are not immune to the observed impacts of including a variable number of “clean frames” across scans (Supplemental Text S2 and Figs. S2–S5). As participants, scans, and scan sessions often differ in their number of clean frames present, this will naturally introduce the biases noted throughout the present report which must be dealt with, irrespective of the data cleaning technique.

### Practical considerations for data acquisition

Older adults (Mowinckel et al. 2012; Van Dijk et al. 2012; Savalia et al. 2017) and children (Power et al. 2014) exhibit greater amounts of head movement relative to younger adults, and many patient groups exhibit greater head movement relative to age-matched control participants (Haller et al. 2014; Pardoe et al. 2016). This means that less clean data are available for these cohorts following preprocessing if a fixed amount of data is collected (even in cases where data are not removed, as with outlier frame regression techniques). Importantly, supplementary analyses indicate that it is the total scan time as opposed to number of frames that drive the reliability effects described in this report (Supplemental Text S4 and Fig. S8). This observation is in line with the properties of resting-state time series that by definition comprise lower-frequency signals (< 0.1 Hz). Using longer scan length can capture more variance from lower-frequency domains and result in better estimation of functional networks. This conclusion is similar to that reached by a previous study, which focused on ICC values of specific connections (Birn et al. 2013). As a result, in order to obtain a sufficient and equivalent amount of data that will allow reliable measurement of RSFC brain network segregation or integration across children, older individuals, and patient populations, data collection efforts in these cohorts will generally need to be longer relative to younger adult cohorts (either via longer duration scans protocols, and/or via real-time monitoring of head motion to increase the likelihood that enough data is collected to obtain reliable estimates of function brain networks; Dosenbach et al. 2017). While newer generations of data acquisition techniques (e.g. multi-echo fMRI; Lynch et al. 2020) or data cleaning methods (e.g. Gratton et al. 2018; Kaplan et al. 2022) may reduce the amount of data needed to achieve high-quality signal, the necessity of equating clean frames across sessions remains.

With very large quantities of data, equating data may be less necessary given that measures eventually asymptote [i.e. the confounding effect is particularly insidious when less data are available for participants (e.g. <=20 min)]. However, many data acquisition protocols and existing datasets (including several large-scale initiatives including UK Biobank, Adolescent Brain Cognitive Development, Alzheimer’s Disease Neuroimaging Initiative, etc.) do not have the luxury of collecting the identified scan lengths given the constraints introduced by scanning special populations (in terms of age and clinical status) and limited amount of scanning time available per participant. As such, while the present results support the value of acquiring greater amounts of data, in many practical situations, it is critical to recognize the importance of equating data quantity toward achieving consistency in brain network measurement.

## Conclusion

Given the increased focus on measures of large-scale brain network organization, it is critical that researchers understand the consistency and reliability of RSFC-based measures of brain network segregation and integration. We have demonstrated that measures of brain system segregation, modularity, mean CC, and mean PC vary with increasing amounts of data for a given individual. With sufficient data, low inter-session variability of network measures can be achieved, indicating that obtaining reliable estimates of these measures is feasible within an individual. Notably, obtaining accurate and reliable measures of segregation and integration necessitates equating the quantity of data included in their calculation across scan sessions (between and/or within individuals). The failure to equate data quantity results in misestimation of cross-sectional differences and within individual changes of brain network changes, which can impact the interpretation of results. In most circumstances then, it is reasonable to assert that while more data is better, equal data is necessary.

## Supplementary Material

Han_SegregationReliability_SI_20240115_bhad506

## Data Availability

The MSC dataset is available via OpenNeuro data repository (https://openneuro.org/datasets/ds000224). The HCP-YA dataset is available via the Human Connectome Project with ConnectomeDB (https://www.humanconnectome.org). The HCP-A dataset is available on the NIMH Data Archive (https://nda.nih.gov/).
