# Supplementary material for: Measures of resting-state brain network segregation and integration vary in relation to data quantity: implications for within and between subject comparisons of functional brain network organization
Source: Cereb Cortex. 2024 Feb 22;34(2):bhad506. doi: 10.1093/cercor/bhad506 (PMC10883417; doi:10.1093/cercor/bhad506)
Supplement: Han_SegregationReliability_SI_20240115_bhad506 [file han_segregationreliability_si_20240115_bhad506.pdf]

Measures of resting-state brain network segregation and integration vary in relation to data quantity: Implications for within and between subject comparisons of functional brain network organization

Supplemental Information

Liang Han[1], Micaela Y. Chan[1], Phillip F. Agres [1],

Ezra Winter-Nelson[1], Ziwei Zhang [1], and Gagan S. Wig[1,2]

[1]Center for Vital Longevity and School of Behavioral and Brain Sciences, University of Texas at Dallas, Dallas, TX, 75235, USA

[2]Department of Psychiatry, University of Texas Southwestern Medical Center, Dallas, TX, 75390, USA

Text S1 Lower modularity ICC values are a consequence of poor participant discriminability
Figure S1 Between-subject and within-subject variability of the network measures
Text S2 Alternate data processing and denoising techniques are also prone to the impact of data characteristics on reliability of network measures
Figure S2 Between-session variability of system segregation using data where interpolated data were removed or included
Figure S3 Between-session variability of segregation is greater with data including interpolated frames than in data without interpolated frames; this magnitude of the difference is related to the amount of data loss due to head motion
Figure S4 Head motion residuals after ICA-FIX denoising process contaminates the estimation of system segregation
Figure S5 'Spike-regression' of high motion frames does not alleviate the confounding effect of having varying number of high motion frames across scans on estimates of brain system segregation
Text S3 The relationship between data quantity and segregation measurement reliability can be attributed to sampling variability
Figure S6 System segregation varies with the amount of gaussian noise added to nodal time-series
Figure S7 System segregation varies with the amount of gaussian noise added to within- and between-system correlations
Text S4 Network measures are more sensitive to the total time duration over which data are sampled than absolute amount of data (number of frames)
Figure S8 The total scan time over which frames are sampled influences estimates of brain system segregation

Text S1: Lower modularity ICC values are a consequence of poor participant discriminability

Intra-class correlation (ICC) is defined from two types of variability: within-cluster (in our case, participant) similarity and between-cluster (participant) variability. Specifically, ICC quantifies the degree to which the data within a cluster is reliable while also estimating the degree to which data between clusters can be discriminated. ICC values range from 0 to 1, with higher ICC values indicating that the data are reliable within clusters and discriminable across clusters. For this reason, a measure can have low within-subject variance but still have poor ICC due to there not being a lot of variability across participants. These two components were plotted for the network measures in the figure below (**Figure S1**).

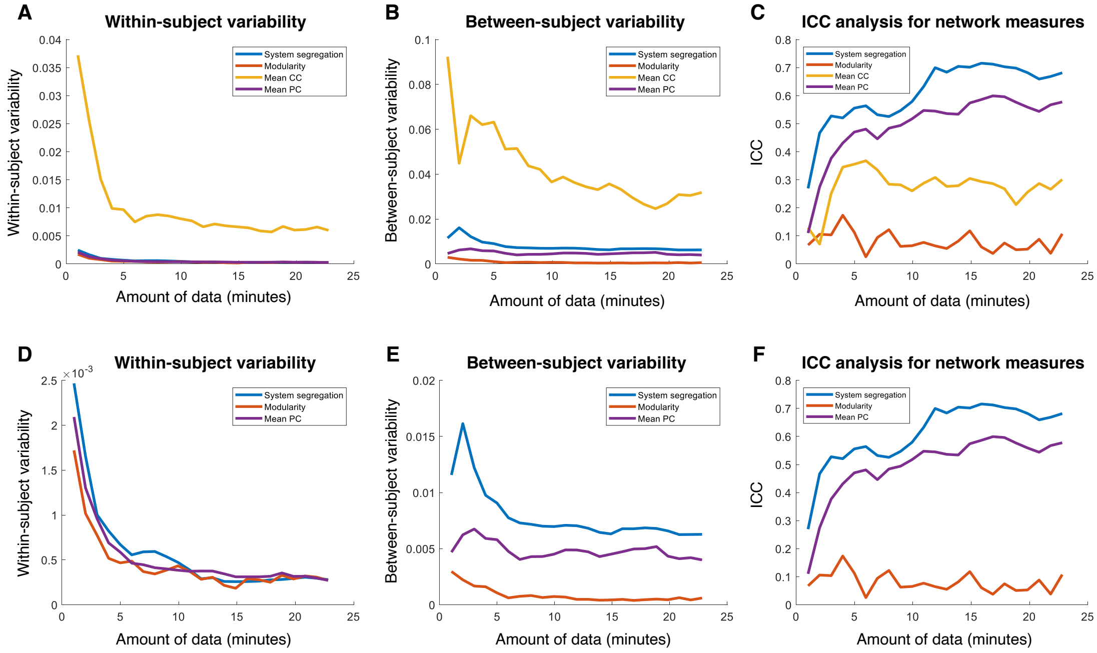

Figure S1. Between-subject and within-subject variability of the network measures. The left panels depict within-subject variability (defined as mean square within participants) of network measures as a function of data quantity (A, D); the middle panels depict between-subject variability (defined as mean square between participants) of the measures (B, E); the right panels depict ICC values as a function of data quantity (C, F). Given the very high values for mean Clustering Coefficient (CC), the bottom row of figures re-plots the variability and ICC measures but without including mean CC, so that values for the other measures can be better appreciated.

As can be appreciated from the figure, modularity has low within-subject variability, as does system segregation and participation coefficient. But the between-subject variability of modularity is also consistently lower in comparison to the other measures. This trend is consistent even when more data are used. Given the absence of discriminability, this leads to lower ICC values of modularity, compared to other network measures, regardless of amount of data. Conceptually, this indicates that modularity may not be an ideal measure for discrimination of participants, even with large data quantities.

Text S2: Alternate data processing and denoising techniques are also prone to the impact of data characteristics on reliability of network measures

1. Effect of including interpolated frames on reliability of network measures.

A take-home point from the present study is that obtaining reliable estimates of network measures requires sufficient amounts of clean data (e.g., ≥ 20 minutes) and equating the number of frames across scans/participants. However, data denoising techniques relying on frame censoring (i.e., 'scrubbing') explicitly remove frames due to greater head movement. One plausible way to mitigate this issue may be to interpolate frames in replacement of high-motion frames, and retain these interpolated frames in the time-series. Interpolated frames are synthetic data derived from surrounding datapoints. These interpolated frames are ultimately censored after data processing, because they are duplications from clean volumes with less head motion and do not add new information to the data (Power et al., 2015). However, including these synthetic data may serve to counteract the presently described biases, particularly for the participants with greater head movement. We test this hypothesis directly.

Using MSC data, we evaluated the impact of retaining interpolated frames as compared to removing them, on between-session variability of system segregation. For this comparison, 15 minutes of clean data with interpolated frames removed (total of 409 frames) were sampled to compute system segregation for each session, and between-session SD of segregation was then estimated for each participant. This was compared to time-series where high movement frames had been identified and removed, but the interpolated frames were retained. Critically, this latter condition also included 15-minutes data [total of 409 frames].

As shown in **Figure S2,** the between-session SD using the data where interpolated frames are removed is nominally lower (M_{SD} = 0.017±0.005) than when interpolated data are retained (M_{SD} = 0.034±0.034), although this difference is not statistically significant using a paired-sample t-test ($t(8)_{removed\text{-}retained} = -1.745$, $p = 0.119$).

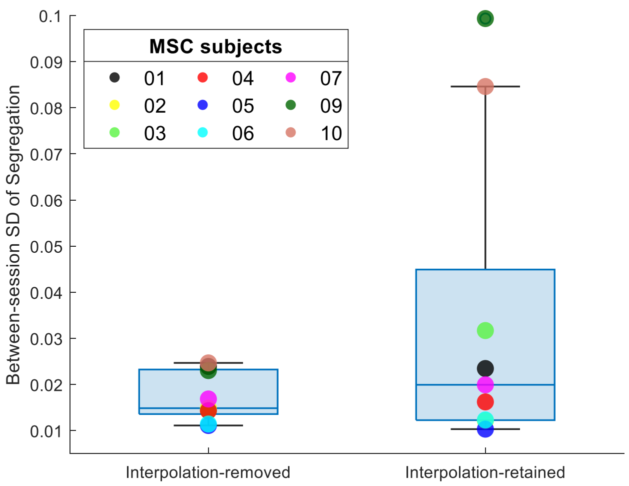

Figure S2. Between-session variability of system segregation using data where interpolated data were removed or included. To compare the impact of retaining interpolated frames as compared to removing these frames, two conditions were designed; sampling 15-minutes of data with interpolated frames removed (Interpolation-removed) and sampling 15-minutes of data without removing the interpolation (Interpolation-retained). System segregation was calculated for each condition and between-session variability of segregation was estimated in each participant and compared between the two conditions. As shown in the figure, the between-session SD when interpolated data are removed (M_{SD} = 0.017±0.005) is lower than the SD values from data including interpolated frames (M_{SD} = 0.034±0.034), although this difference is not statistically significant (see text).

This result demonstrates that including interpolated frames leads to potentially greater between-session variability than using data where these frames have been removed, indicating it is not advisable to retain these frames. The non-significant relationship may also be attributed to fewer data points available for the comparison (9 participants), and less frame loss due to minimal head motion in some participants. Consistent with this latter idea, participants with greater head-movement associated frame loss exhibit greater differences of between-session SD when comparing system segregation from scans where their interpolated data was removed to where interpolated volumes were retained. Specifically, the difference of between-session SD was computed for each participant (between-session $SD_{retained}$ – between-session $SD_{removed}$), then correlated to data loss rate across all participants. The result indicates that data loss rate is significantly correlated with differences of between-session SD ($r = 0.875$, $p = 0.002$, **Figure S3**). This indicates that some participants lose more data due to greater head motion, and retaining their interpolated data results in greater between-session variability as compared to when the data are removed.

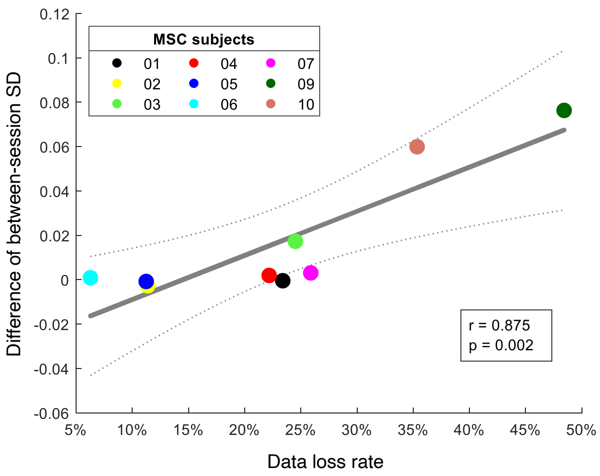

Figure S3. Between-session variability of segregation is greater with data including interpolated frames than in data without interpolated frames; this magnitude of the difference is related to the amount of data loss due to head motion. Difference of between-session variability was calculated for each participant (between-session $SD_{retained}$ - between-session $SD_{removed}$). This difference is positively related to data loss rate ($r = 0.875$, $p = 0.002$), which indicates that participants with more data loss due to greater head motion are more likely to have greater differences in between-session variability when interpolated frames are included as opposed to retained.

2. Effect of alternate data cleaning/pre-processing techniques on reliability of network measures.

In addition to data censoring ('scrubbing'), there exist a variety of processing techniques to denoise resting-state data which do not explicitly remove data frames, and thus may be less sensitive to the presently observed biases. We evaluate examples from two of classes of these techniques here: ICA-based methods and regression-based methods.

To evaluate the effectiveness of ICA-related denoising methods which do not explicitly remove data, we utilized the HCP-YA dataset, which has undergone ICA-FIX (FMRIB's ICA-based X-noiseifier; Salimi-Khorshidi et al., 2014) processing. This dataset enabled us to assess system segregation across varying levels of head motion but with alternate processing strategies that do not explicitly remove high-motion frames. We hypothesized that inclusion of differing amounts of high movement frames should systematically bias system segregation estimates across participants (even though these frames are not removed as is done with data scrubbing).

For each HCP-YA participant, frame displacement (FD) was estimated prior to any head motion correction. Subsequently, the absolute difference between mean FDs of session 1 and session 2 were calculated. Based on this summarized head motion difference, two groups of participants were identified. Participants (N=100) exhibiting higher differences in FD across their scanning sessions, relative to the other participants, were categorized as the "high FD difference" group ($M_{FDdiff} = 0.0118 \pm 0.0052$). Conversely, participants (N=100) exhibiting lower differences in FD across their scanning sessions, relative to the other participants, were categorized as the "low FD difference" group ($M_{FDdiff} = 0.0003 \pm 0.0002$; two-sample t-test: $t(198) = 22.11$, $p < 0.001$). In each participant, system segregation was calculated for each session using all the resting-state data that had undergone ICA-FIX preprocessing in the HCP pipeline. Absolute difference of segregation between the 2 sessions was calculated for each participant, and then used to compare the two groups using a two-sample t-test.

As shown in **Figure S4**, between-session difference of system segregation is greater in the "high FD difference" group ($M_{SegDiff} = 0.089 \pm 0.059$) relative to the "low FD difference" group ($M_{SegDiff} = 0.050 \pm 0.042$; two-sample t-test: $t(198) = 5.37$, $p < 0.001$). This indicates that even after ICA-FIX denoising, having scans that vary in terms of original data quality as defined by head movement (and presumably, residual head motion) systematically biases the estimation of system segregation and inflates the between-session variability of this measure, paralleling the impact of having variable data quantity across scans.

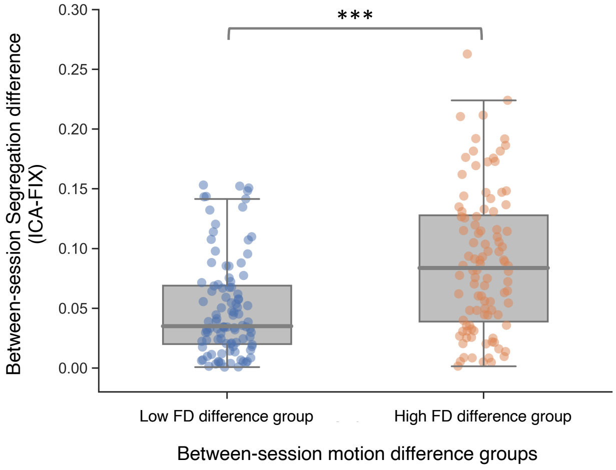

Figure S4. Head motion residuals after ICA-FIX denoising process contaminates the estimation of system segregation. Two groups of subjects were identified based on the absolute difference between mean head motions estimates (measured by frame displacement [FD]) of session 1 and session 2. The first group exhibited higher between-session difference in FD among all subjects, while the second group exhibited lower between-session difference in FD. System segregation was calculated using all ICA-FIX processed data in each session. Absolute difference in segregation between the 2 sessions was calculated for each subject, and then used to compare the two groups using a two-sample t-test. The between-session difference in segregation in the group with higher between-session difference in FD ($M_{SegDiff}= 0.089\pm0.059$) is greater relative to the group of lower FD difference ($M_{SegDiff} = 0.050\pm0.042$; two-sample t-test: $t(198) = 5.37$, $p < 0.001$). This indicates that within subject differences in head motion residuals across sessions lead to confounds in estimation of system segregation, analogous to that observed with varying frame amounts. Note: *** $p < 0.001$.

Another common way to account for head motion in scans is to mark frames with excessive head motion, and then include 'spike' regressors of this time series (e.g., Lemieux et al., 2007; Satterthwaite et al., 2013; Siegel et al., 2014). We employed a comparable strategy to the existing works and obtained the residual time series in each participant, from which system segregation values were calculated. Specifically, instead of going through data scrubbing, temporal masks that marked frames with excessive head motions were used as a regressor, based on which the residual resting-state time series was obtained and used to calculate correlation matrices and measure system segregation for each participant. This approach did not help to minimize the confounds of having varying number of clean frames across sessions; participants with greater between-session differences in high head motion frames exhibited greater between-session differences in segregation (two-sample t-test: $t(198) = 5.43$, $p < 0.001$; **Figure S5**).

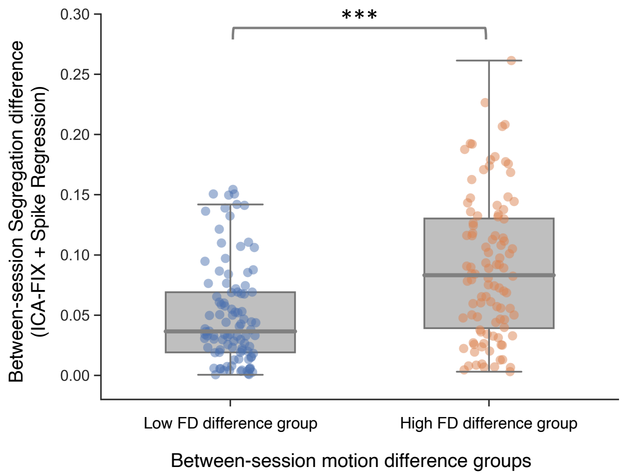

Figure S5. 'Spike-regression' of high motion frames does not alleviate the confounding effect of having varying number of high motion frames across scans on estimates of brain system segregation. Following our standard workflow of resting-state data processing (but without including data scrubbing), temporal masks that marked frames with excessive head motions were used as a regressor and the residual resting-state time series was used to calculate correlation matrices and measure system segregation for each participant. Participants with greater between-session difference in number of high motion frames exhibited greater between-session difference in segregation. Note: *** $p < 0.001$.

Text S3: The relationship between data quantity and segregation measurement reliability can be attributed to sampling variability

RSFC-based network measures vary in relation to data quantity. This observation may be understood from the perspective of sampling error: using lower quantities of data could lead to greater sampling error that results in misestimation of functional networks and corresponding measures that describe their organization. To more directly evaluate this hypothesis, an additional analysis was conducted in which noise was directly added to the resting-state data, in order to simulate the scenario of high sampling error in the data. Specifically, white Gaussian noise was sampled and added to every node's time series for each participant. For each node, a noise vector was created that had the same number of data points as the node's resting-state time series in each session; this noise vector followed a normal distribution with a mean of zero. Next, the noise vector was added to the node's time series. Using this noise-added time-series, cross-correlation matrices were computed and system segregation was calculated for the participant. This procedure was repeated 1000 times with increasing variability of noise in each iteration (i.e., the temporal SD of the noise vector increased from 0.1 to 100), which varied the amount of noise added to the time series.

As shown in the figure below, system segregation changes as a function of added noise (variability). Specifically, segregation is lower when the noise added to the time series is greater (higher temporal SD).

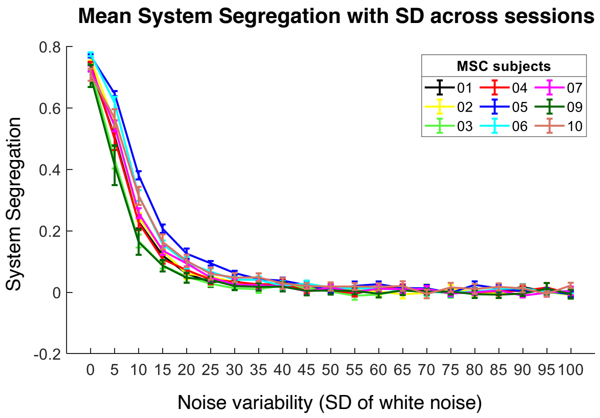

Figure S6. System segregation varies with the amount of gaussian noise added to nodal time-series. For each participant, white Gaussian noise with different amounts of temporal variability was created and added to the resting-state time series of every node's time series to evaluate the effect of signal noise levels on estimation of resting-state system segregation. This procedure was repeated for all available sessions of the participant. The figure demonstrates that as the level of noise variability increases (i.e., higher temporal SD, x-axis), system segregation decreases and approaches a value of 0. When the SD of the noise = 0, the segregation values correspond to correlation matrices built from the original time series. In all cases, the amount of data (frames) contributing to the measure has been equated (using all available clean frames for each participant). Each line represents the mean segregation across sessions for each participant. Error bars are the standard deviation across sessions.

This relationship appears similar to how segregation varies as a function of data quantity, and provides a plausible explanation as to why segregation varies with amount of data. Lower quantities of data could increase sampling variability (akin to the presence of higher temporal SD). The presence of this sampling variability confounds the estimation of edges and network organization (measured by segregation).

Next, the analyses above were repeated, but the introduction of time-series noise was limited to either within-system or between-system correlations. The results indicate that system segregation decreases when noise is added specifically to the time series relationships corresponding to within-system correlations (**Figure S7A**), but increases when noise is added specifically to the time series relationships corresponding to between-system correlations (**Figure S7B**). Based on the observations, greater noise in the resting-state time series likely explains the relationship between system segregation and the amount of data due to random effect of sampling variability, particularly on within-system correlations, with insufficient data.

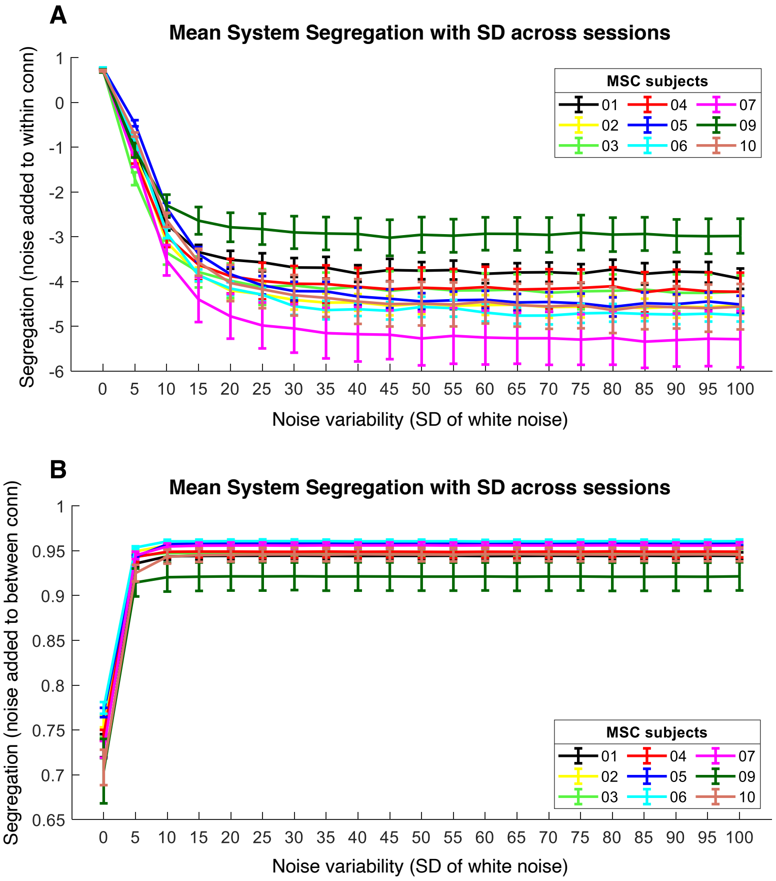

Figure S7. System segregation varies with the amount of gaussian noise added to within- and between-system correlations. White gaussian noise with different amounts of temporal variability was added specifically to the time series used to compute either A) within-system correlations, or B) between-system correlations. The variation of system segregation as a function of amount of noise exhibits distinct patterns across these two procedures. When noise is added to within-system correlations, system segregation increases with increasing noise, which is similar to the

effect observed when noise is added uniformly to all nodal time-series or when limiting the amount of data used to compute the measure. In contrast, when noise is added to between-system correlations, system segregation increases with increasing noise. These results suggest that variation of system segregation as a function of noise levels are mainly driven by altered within-system correlations.

Text S4: Network measures are more sensitive to the total time duration over which data are sampled than absolute amount of data (number of frames)

The total scan time and the total number of frames are often highly correlated, although important differences may exist. For instance, two data acquisition protocols may differ in their TR (and thus have differing number of total frames), yet have comparable scan time, or vice versa.

Using the HCP-YA dataset, three conditions were designed to sample the resting-state data and calculate system segregation, enabling manipulation of scan length and frame amount by holding the other variable constant: (1) A longer scan length and original frame number condition. In this condition all clean frames from the first 20 min data (1667 frames) for each participant were used to calculate the correlation matrix and system segregation scores. (2) A longer scan length and lesser frame number condition. In this condition, the timeseries from condition (1) scans were down-sampled by selecting every other frame (1st, 3rd, 5th frame, …), to simulate the effect of having a longer TR (original TR [0.72 s] × 2 = current TR [1.44 s]) while maintaining the total length of the time over which the timeseries was collected (833 frames, 20min). As such, in this case, the total number of frames was half of that in condition (1). (3) A shorter scan length and lesser frame number condition. In this condition, the time series from the first 10 minutes of condition (1) scans were sampled. As such, the total length of time over which the timeseries was collected was half of that generated for the matrices in condition (1), but the total number of frames (833 frames) was equivalent to matrices generated in condition (2).

The results are depicted in **Figure S8** and demonstrate that segregation using 20-minutes of data ($M_{segregation} = 0.7367 \pm 0.028$) is comparable to that using 20-minutes of down-sampled data ($M_{segregation} = 0.7366 \pm 0.028$; it was confirmed that the two sets of values are indeed that close), and as expected the difference is not statistically significant (two-sample t-test: $t(1460)_{20all\_vs\_20downsampled} = 0.008$, $p = 0.993$). This indicates that despite having half the number of frames, maintaining the total scan length over which frames are acquired results in comparable estimates of segregation as using all the available data.

Further, system segregation values from both conditions are significantly higher than when shorter scan length and lesser frame numbers are used (10-minute consecutive data; $M_{segregation} = 0.71 \pm 0.034$). Critically, the number of frames included in the 20-minute down-sampled condition (condition (2)) is equivalent to the 10-minute condition (condition (3)), yet system segregation values are significantly higher in the 20-minute down-sampled condition (two-sample t-test: $t(1460)_{20downsampled\_vs\_10all} = 16.91$, $p < 0.001$). This supports the conclusion that total scan time over which data are sampled appears to also be an important factor that must be considered in comparisons.

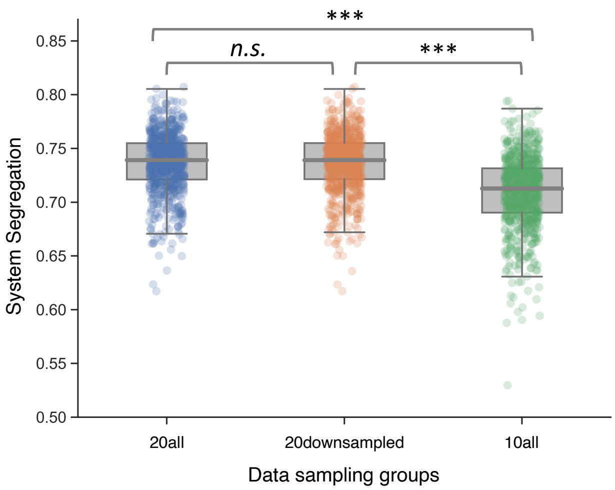

Figure S8. The total scan time over which frames are sampled influences estimates of brain system segregation. Three experimental conditions were designed to sample the resting-state time-series and calculate system segregation, while independently manipulating total scan length versus total frame amount: i) sampling from a longer scan length with all available frames (20-minute time-series, all frames [20all]), ii) sampling from a longer scan length but using lesser frames, to simulate longer TRs (20-minute time-series, every other frame sampled [20downsampled]), and iii) sampling from a shorter scan length with all available frames (10-minute time-series, all frames [10all]). Importantly, the last condition corresponded to an equivalent number of frames as condition ii). There is no significant difference of segregation between 20all and 20downsampled groups ($t(1460)_{20all\text{-}20downsampled} = 0.008$, $p = 0.993$), indicating that if total scan length is preserved, the number of frames may be less critical (at least with the down-sampling parameter used here) towards adequately capturing resting-state variance and computing resultant network measures. In contrast, system segregation of 10all condition is lower than the segregation values from 20all condition ($t(1460)_{20all\text{-}10all} = 16.92$, $p < 0.001$). More importantly, even when frame numbers are equivalent, segregation of 10all is significantly lower than values calculated using the 20downsampled condition ($t(1460)_{20downsampled\text{-}10all} = 16.91$, $p < 0.001$). These findings altogether reveal that total scan time over which data are sampled is an important variable that contributes to precise estimation of system segregation. Note: *** $p < 0.001$; *n.s.*, not significant.

REFERENCES

Lemieux, L., Salek-Haddadi, A., Lund, T.E., Laufs, H., Carmichael, D., 2007. Modelling large motion events in fMRI studies of patients with epilepsy. Magn Reson Imaging 25, 894-901.

Power, J.D., Schlaggar, B.L., Petersen, S.E., 2015. Recent progress and outstanding issues in motion correction in resting state fMRI. Neuroimage 105, 536-551.

Salimi-Khorshidi, G., Douaud, G., Beckmann, C.F., Glasser, M.F., Griffanti, L., Smith, S.M., 2014. Automatic denoising of functional MRI data: combining independent component analysis and hierarchical fusion of classifiers. Neuroimage 90, 449-468.

Satterthwaite, T.D., Elliott, M.A., Gerraty, R.T., Ruparel, K., Loughead, J., Calkins, M.E., Eickhoff, S.B., Hakonarson, H., Gur, R.C., Gur, R.E., Wolf, D.H., 2013. An improved framework for confound regression and filtering for control of motion artifact in the preprocessing of resting-state functional connectivity data. Neuroimage 64, 240-256.

Siegel, J.S., Power, J.D., Dubis, J.W., Vogel, A.C., Church, J.A., Schlaggar, B.L., Petersen, S.E., 2014. Statistical improvements in functional magnetic resonance imaging analyses produced by censoring high-motion data points. Hum Brain Mapp 35, 1981-1996.
